# The social brain network in 22q11.2 deletion syndrome: a diffusion tensor imaging study

**DOI:** 10.1186/s12993-017-0122-7

**Published:** 2017-02-16

**Authors:** Amy K. Olszewski, Zora Kikinis, Christie S. Gonzalez, Ioana L. Coman, Nikolaos Makris, Xue Gong, Yogesh Rathi, Anni Zhu, Kevin M. Antshel, Wanda Fremont, Marek R. Kubicki, Sylvain Bouix, Martha E. Shenton, Wendy R. Kates

**Affiliations:** 1 Department of Psychiatry, SUNY Upstate Medical University, 750 E. Adams St., Syracuse, NY 13210 USA; 2 Department of Psychiatry, Brigham and Women’s Hospital, Harvard Medical School, Boston, MA USA; 3 Syracuse University, Syracuse, NY USA; 4 Departments of Psychiatry and Neurology, Massachusetts General Hospital, Harvard Medical School, Boston, MA USA; 5 Department of Radiology, Brigham and Women’s Hospital, Harvard Medical School, Boston, MA USA; 6 VA Boston Healthcare System, Harvard Medical School, Brockton, MA USA

**Keywords:** 22q11.2 deletion syndrome, Social brain network, Social cognition, Two-tensor tractography, White matter tracts

## Abstract

**Background:**

Chromosome 22q11.2 deletion syndrome (22q11.2DS) is a neurogenetic disorder that is associated with a 25-fold increase in schizophrenia. Both individuals with 22q11.2DS and those with schizophrenia present with social cognitive deficits, which are putatively subserved by a network of brain regions that are involved in the processing of social cognitive information. This study used two-tensor tractography to examine the white matter tracts believed to underlie the social brain network in a group of 57 young adults with 22q11.2DS compared to 30 unaffected controls.

**Results:**

Results indicated that relative to controls, participants with 22q11.2DS showed significant differences in several DTI metrics within the inferior fronto-occipital fasciculus, cingulum bundle, thalamo-frontal tract, and inferior longitudinal fasciculus. In addition, participants with 22q11.2DS showed significant differences in scores on measures of social cognition, including the Social Responsiveness Scale and Trait Emotional Intelligence Questionnaire. Further analyses among individuals with 22q11.2DS demonstrated an association between DTI metrics and positive and negative symptoms of psychosis, as well as differentiation between individuals with 22q11.2DS and overt psychosis, relative to those with positive prodromal symptoms or no psychosis.

**Conclusions:**

Findings suggest that white matter disruption, specifically disrupted axonal coherence in the right inferior fronto-occipital fasciculus, may be a biomarker for social cognitive difficulties and psychosis in individuals with 22q11.2DS.

**Electronic supplementary material:**

The online version of this article (doi:10.1186/s12993-017-0122-7) contains supplementary material, which is available to authorized users.

## Background

Chromosome 22q11.2 deletion syndrome (22q11.2DS), also known as velo-cardio-facial syndrome (VCFS), or DiGeorge syndrome, is a genetic neurodevelopmental disorder that occurs as a result of an interstitial deletion of 40–50 genes on the long arm of chromosome 22 [1]. The most recent estimate of the syndrome’s incidence is 1:992 live births [2]. The deletion is associated with a myriad of physical features, including distinctive facial characteristics, palatal abnormalities, and cardiac anomalies [3]. In addition to these physical characteristics, individuals with 22q11.2DS often possess a distinct neuropsychological profile, consisting of a full scale IQ in the borderline range, as well as deficits in executive function, working memory, and visuospatial abilities [4–6]. 22q11.2DS is also associated with multiple psychiatric comorbidities, including mood disorders [7–10], anxiety disorders [3, 9, 11–13], attention-deficit/hyperactivity disorder (ADHD; [9–11, 13]), autism spectrum disorder (ASD; [5, 14, 15]), and, in up to 30–40% of adults, psychotic disorders such as schizophrenia [16, 17]. In fact, aside from having a monozygotic twin with schizophrenia, 22q11.2DS is the next highest risk for developing schizophrenia [16].

### Social processing and the social brain

Many individuals with 22q11.2DS also experience social difficulties, including shyness, withdrawal, social immaturity, and deficits in social cognition [18–21]. Social cognition refers to the mental processes that subserve social interactions [22] and includes theory of mind, i.e., being able to see things from another’s perspective, also referred to as “mentalizing skills,” attributional style, social perception, and emotional processing abilities [22, 23]. Importantly, social cognitive impairments have also been identified in individuals with schizophrenia [24] and autism spectrum disorders [25].

Of further note, functional brain imaging studies have identified two major social networks that are involved in social cognition tasks: a mirror network, which is involved in reading another individual’s body language, and a mentalizing network that allows for social mentalizing, or taking another’s perspective [26–28]. These networks involve several brain regions in the prefrontal (i.e., dorsolateral-ventrolateral-medial prefrontal and premotor cortices), pregenual and dorsal anterior cingulate regions, the insula, the amygdala, inferior parietal lobule and precuneus as well as the temporopolar and the temporo-parieto-occipital junction areas [24, 29, 30]. Insofar as studies have shown associations between aberrant connectivity within the mentalizing network and social cognitive difficulties among individuals with schizophrenia [31–34], ASD [35–38], and 22q11.2DS [39, 40], our primary interest was to examine the white matter tracts underlying social cognition in 22q11.2DS.

### Structural imaging: diffusion tensor imaging

Recent advances in imaging, including diffusion tensor imaging (DTI) and fiber tractography post-processing analyses, have enabled the use of noninvasive methods to measure structural white matter tract integrity in vivo by examining the diffusion of water molecules in the brain. DTI metrics, including fractional anisotropy (FA), a measure of white matter integrity, radial diffusivity (RD), a purported measure of myelin integrity, and axial diffusivity (AD), a purported measure of axonal integrity, make it possible to quantify differences in these metrics across groups. Studies in individuals with 22q11.2DS show altered white matter microstructure in long-range and limbic connections (see review by [41]), including fronto-parietal, fronto-temporal, and parieto-occipital networks, as well as in cingulum bundle (CB), anterior limb of the internal capsule (ALIC), anterior thalamic radiation and uncinate fasciculus (UF), all areas known to show abnormalities in individuals with schizophrenia [41–46].

#### DTI tracts related to social functioning

Several white matter tracts are believed to play important roles in the transmission of social information. For example, the CB is an associative bundle of fibers running through the cingulate gyrus around the corpus callosum. Longer fibers run from the anterior temporal gyrus to the orbitofrontal cortex, while shorter fibers connect the four lobar regions of the brain and the cingulate cortex [47]. The location of the CB within the limbic system suggests that it plays an important role in emotional information processing. The UF is also part of the limbic system, and connects the amygdala to the anterior temporal lobe and orbitofrontal cortex [47]. Thus, the UF is also believed to play a role in emotion processing.

The superior longitudinal fasciculus (SLF) is composed of four separate components, SLF I—SLF III and the arcuate fasciculus. The SLF II composes the central core of white matter above the insula, and is believed to be the major link between the parietal lobe and prefrontal cortex, thereby implicating a role in the perception of visual space [48]. The inferior fronto-occipital fasciculus (IFOF) connects the ventral occipital lobe to the orbitofrontal cortex, and is involved in visual processing/facial emotion recognition [47]. A study by DeRosse and colleagues [49] found that lower FA in the IFOF is related to higher levels of schizotypy, indicating a possible role for IFOF in experience/affect sharing [49]. The inferior longitudinal fasciculus (ILF) connects the occipital and temporal lobes. While not much is known regarding the ILF’s functionality, it is believed to be involved in face recognition and visual object perception [47, 50, 51]. Finally, the anterior thalamic radiation (ATR) connects the anterior nuclear and midline nuclear groups of the thalamus with the frontal lobe [52]. The ATR is located in the thalamolimbic area, and hypoconnectivity of the ATR has been demonstrated in males with ASD, a disorder associated with social cognitive impairment [53]. Studies have also shown that functional connectivity (i.e., fMRI) reflects structural connectivity (i.e., DTI) in the mentalizing network [54, 55].

The aim of the current project was to explore structural DTI tracts hypothesized to be involved in the social brain, as well as to investigate associations with measures of social and emotional processing. We predicted that there would be significant differences between individuals with 22q11.2DS and controls in DTI metrics, including FA, RD, AD, and the number of streamlines (an estimate of fiber bundles). Following the expected reductions in DTI metrics, we also hypothesized: (1) FA in IFOF, SLF, the thalamo-frontal tract, and CB would be correlated with a measure of social responsiveness; (2) FA in ILF and IFOF would be associated with a measure of experience and affect sharing; and (3) FA in UF and IFOF would be associated with emotion regulation and cognitive reappraisal. Finally, we predicted that alterations in DTI metrics in 22q11.2DS would be associated with a dimensional measure of both positive and negative symptoms of prodromal/overt psychosis.

## Methods

### Participants

The imaging and psychiatric data presented in this study were derived from a subsample of participants enrolled in a longitudinal study of risk factors for psychosis in 22q11.2DS [56]. This subsample consists of 57 participants with 22q11.2DS, 12 unaffected siblings, and 18 community controls who returned for the fourth time point of the study. Participants were recruited from the International Center for Evaluation, Treatment, and Study of Velo-Cardio-Facial Syndrome at SUNY Upstate Medical University, parent support groups, and the surrounding community. Presence of the 22q11.2 deletion was confirmed with fluorescence in situ hybridization (FISH). Informed consent was obtained under protocols approved by the medical center’s institutional review board. Initial statistical analyses comparing sibling and community controls did not differ for any of the measures utilized in this study (see Additional file 1). Therefore, we combined the sibling and community controls into one control group for the remainder of the analyses. Demographic information is provided in Table 1.

**Table 1:** Participant demographics

Demographic variable	22q11.2DS (N = 57)	Control (N = 30)
Age [Mean (SD)]	20.87 (2.29)	20.97 (1.46)
Gender [N (%)]		
Male	31 (54.4)	17 (56.7)
Female	26 (45.6)	13 (43.3)
FSIQ [Mean (SD)]	74.54 (11.82)***	109.47 (16.02)
Race [N (%)]		
Native American	0	1 (3.3)
Asian	1 (1.8)	2 (6.7)
African American	0	1 (3.3)
Caucasian	51 (89.5)	24 (80.0)
More than one	2 (3.5)	1 (3.3)
Unknown	3 (5.3)	1 (3.3)
Psychiatric diagnosis [N (%)]		
Mood disorder	7 (12.3)	1 (3.3)
Anxiety disorder	16 (28.1)	5 (16.7)
ASD	7 (12.3)	0
ADHD	9 (15.8)	5 (16.7)
Psychotic disorder	4 (7.0)	0
Other	3 (5.2)	0
Any psychiatric diagnosis	27 (47.4)	7 (23.3)

22q11.2DS, 22q11.2 deletion syndrome; FSIQ, full scale IQ; ASD, autism spectrum disorder; ADHD, attention-deficit/hyperactivity disorder

*** *p* < .0001

Several papers examining white matter microstructure have been published on this cohort based on assessment at the third time point of the study (when they were between the ages of 15 and 21) [43, 45, 46]. However, this is the first paper to examine white matter microstructure based on participants’ assessments at the 4th timepoint of the study, when they were between the ages of 18 and 24 years, representing the age window at which this cohort is at highest risk for developing psychotic symptoms. Moreover, whereas previous papers have been based on imaging data acquired from a 1.5 Tesla scanner, the current study is based on data acquired from a 3 Tesla scanner, utilizing state-of-the-art two-tensor tractography to measure white matter microstructure.

Exclusion criteria for all participants included: presence of a seizure disorder, fetal exposure to alcohol or drugs, parent-reported elevated lead levels, birthweight under 2500 g, history of loss of consciousness lasting longer than 15 min, paramagnetic implants, or orthodontic braces. Potential community control participants were also excluded if there was a personal or family history of schizophrenia or bipolar disorder [57]. All participants were screened for psychiatric disorders using the structured clinical interview for DSM-IV-TR (SCID).

### Procedures

As described in previous studies [7, 11, 14, 58], each participant and parent/caregiver completed measures of cognitive and/or social, emotional, and behavioral functioning. All diagnostic interviews were completed by a licensed psychiatrist or psychologist, and all neuropsychological measures were administered by an experienced doctoral-level examiner. Due to the facial features characteristic of 22q11.2DS, evaluator blindness to group assignment was not possible. A licensed psychologist or trained student assistant familiar with the measures double scored all protocols to ensure scoring accuracy. Caregivers completed behavior rating scales and background information while the children and adolescents were completing neuropsychological measures.

### Measures

#### Wechsler Adult Intelligence Scale, third edition (WAIS-III)

The WAIS-III [59] is a test of cognitive ability that provides intelligence quotient (IQ) scores, including a full scale IQ (FSIQ), verbal IQ (VIQ), and performance IQ (PIQ) for individuals 16 years of age and older. The WAIS-III consists of several subtests (mean = 10, SD = 3), which measure various domains. The WAIS-III has outstanding reliability, with internal consistency reliability coefficients at or above .93 for the WAIS-III FSIQ, VIQ, and PIQ [60].

#### Social Responsiveness Scale (SRS)

The SRS is a 65-item parent report questionnaire designed to assess the different dimensions of interpersonal behavior, communication, and repetitive/stereotypic behaviors that are characteristic of autism spectrum disorders [61]. Psychometric properties of the SRS are excellent, with total score alpha coefficients above .90 for both males and females in both clinical and normative sample [61]. In this study, we used the adult research version of the SRS to assess for social deficits, with higher total raw scores indicative of more severe social impairment [62].

#### Junior Schizotypy Scale (JSS)

The JSS is a 50-item self-report questionnaire used to measure schizotypal personality traits in adolescents, which are believed to indicate a predisposition to schizophrenia in adulthood. The JSS provides scores for five subscales, each of which reflect a particular aspect of schizotypy: cognitive, perceptual, social, impulsive nonconformity, and physical anhedonia [63]. Higher scores on each scale indicate higher levels of schizotypy.

#### Trait Emotional Intelligence Questionnaire (TEIQue)

The TEIQue [64] is a 153-item questionnaire based on trait emotion intelligence theory. The TEIQue uses a 7-point Likert response scale (1: disagree completely; 7: agree completely) to measure the level of various facets of emotional intelligence. Higher scores on each facet represent better levels of perceived abilities and dispositions [64]. This study used the parent version of the TEIQue, in which the parent or caregiver rated the participant’s emotional intelligence. For the current study, we were particularly interested in the four facets that relate to social cognition; therefore, our analyses focused on emotion regulation (the degree to which an individual has control over his or her emotions), empathy (the ability to take another’s perspective), social awareness (social skills and the ability to adapt to and interact in various social situations), and emotion perception (the ability to perceive one’s own and others’ emotions).

#### Structured interview for prodromal syndromes (SIPS)

The SIPS [65] is a scale that measures the severity and change of individuals who are experiencing pre-psychotic symptoms. The SIPS consists of five positive symptom items, six negative symptom items, four items related to disorganized symptoms, and four general symptom items, each of which are rated on a severity scale ranging from 0 (never or absent) to 6 (severe and psychotic, or extreme). The SIPS was administered to all study participants and separately to their parents. The positive symptoms (SIPS PS) and negative symptoms (SIPS NS) scores were used for the purpose of determining prodromal/psychotic symptoms.

### MRI acquisition/DTI processing

#### Scan acquisitions

For the time point examined in this study (Time Four), images were acquired using a 3T Siemens Magnetom Tim Trio scanner (Siemens Medical Solutions, Erlangen, Germany).The high resolution anatomic scan consisted of an ultrafast gradient echo 3D sequence (MPRAGE) with PAT k-space-based algorithm GRAPPA. The parameters included: echo time = 3.31 ms; repetition time = 2530 ms; matrix size = 256 × 256; field of view (FOV) = 256 mm; slice thickness = 1 mm. The DWI sequence consisted of 64 transverse slices with no gaps and 2.0 mm nominal isotropic resolution (TR/TE = 8600/93 ms, FOV = 244 × 244, data matrix = 96 × 96, zero-filled and reconstructed to 256 × 256). Diffusion weighting was applied along 64 directions with a ***b*** factor = 700 s/mm^2^. One minimally weighted volume (b_0_) was acquired within each DWI dataset. The total scan time to acquire the DWI dataset was 4 min., 52 s. A high resolution T2 scan was also obtained to align with the DWI images.

#### Diffusion tensor imaging preprocessing

An in-house script was used to correct for eddy current distortions and head motion. This script registered each diffusion-weighted volume to the baseline volume using FSL (http://fsl.fmrib.ox.ac.uk) linear registration software “FLIRT”. Motion correction was not performed.

#### Whole brain tractography

For the purposes of this study, we used two-tensor tractography to determine white matter tracts/bundles. As compared to single tensor tractography, two-tensor tractography offers a better fiber representation in both fiber branching and fiber crossing by computing two tensors for each voxel [66]. We generated fiber tracts from DWI images using the Unscented Kalman Filter (UKF) based on two-tensor tractography algorithm [67]. Tract seeding was completed in every voxel where the primary single tensor FA value was larger than .18, with each voxel seeded 10 times. Fibers between neighboring voxels were traced following the direction of the primary tensor component. Fibers were terminated when the primary tensor FA value was less than .15.

#### FreeSurfer parcellations and registration to DTI space

We used FreeSurfer software (http://surfer.nmr.mgh.harvard.edu) to obtain regions of interest via an automated approach, which parcellated the cortical and white matter regions. We applied FreeSurfer software to segment T1-weighted SPGR images into 34 bilateral, cortical and white matter regions for each participant [68]. The label map with FreeSurfer-generated regions of interest was registered to the DWI space by first diffeomorphically registering a T2 image in the same space as the SPGR image to the baseline DWI image of the same participant using the FLIRT algorithm of the FSL software [69], and then applying this diffeomorphism to register the FreeSurfer-generated label map to the DWI space for the same participant. We performed this transformation of the FreeSurfer label map to DWI space for each participant.

#### White matter query language

White matter query language (WMQL) was used to extract fiber tracts from the two-tensor whole brain tractography [70]. WMQL was designed to use neuroanatomical definitions of white matter to estimate fiber tracts [47]. Fiber tract definitions were based on cortical regions known to be connected via these fiber bundles, as well as on white matter regions where the fiber tract is expected to project. These definitions used the FreeSurfer-generated parcellations of cortical and white matter regions [70]. We implemented WMQL queries to extract the left and right hemisphere CB, ILF, SLF II, and thalamo-frontal tracts. Because the WMQL approach does not rely on a specific atlas, label maps other than those generated by FreeSurfer can also be used. We extracted the right and left hemisphere UF and IFOF from the 2-tensor whole brain tractography using a label map with manually drawn ROIs. The DTI metrics of FA, AD, and RD were extracted from the entire fiber tract and the mean values were computed. WMQL also allowed us to calculate the number of streamlines for each tract, and only tracts with more than 10 streamlines were reported. Two-tensor tractography was first used in a study on first-episode schizophrenia [71], and in later studies combining two-tensor tractography with WMQL queries [48, 70]. It has not, until this study, been used in 22q11.2DS.

### Statistical analyses

The data were examined for normality in order to ensure they met criteria for the assumptions of statistical tests to be used. For variables that did not meet the assumption of normality (i.e., skewness and/or kurtosis <1.0), we applied a log transformation to normalize the data. For data that remained nonnormal after transformation, we created standardized residuals so that nonparametric tests could be used. Where appropriate, analysis of covariance (ANCOVA) and multivariate analyses of covariance (MANCOVAs), using either hemispheric white matter volume or FSIQ as covariates, were used to investigate possible differences between groups on DTI metrics and social brain measures, respectively. Follow up ANOVAs examined which dependent variables drove the significant differences in DTI metrics and scores on social brain measures. In the Results section, we report on DTI metrics that passed Bonferroni correction for multiple comparisons on each hemispheric tract, as well as subscales of social brain measures that passed Bonferroni correction for each measure. Individual Bonferroni correction thresholds varied according to the number of dependent variables within each measure. We also used Pearson correlations to examine relationships between significantly different DTI tract metrics and social brain measures. We used Spearman correlations for variables that remained nonnormal after log transformation. Finally, we used zero-inflated Poisson (ZIP) regressions to analyze the associations between social measures and positive prodromal symptoms, and DTI tracts and positive prodromal symptoms. All data were analyzed using SPSS v. 23 or Stata v. 12.0.

## Results

### DTI tract differences

To explore group differences in DTI metrics, we conducted a multivariate analysis of covariance (MANCOVA), using hemispheric white matter volume as a covariate, to compare participants with 22q11.2DS and controls. Table 2 presents the descriptive statistics for all DTI metrics, and Table 3 includes results of the MANCOVA. Compared to controls, participants with 22q11.2DS showed a significant increase in left FA, suggesting increased white matter integrity, and a significant decrease in left RD (suggesting decreased white matter integrity) within the IFOF; a significant increase in right hemisphere FA (increased white matter integrity) and a significant decrease in right RD (decreased white matter integrity) within the CB; a significant increase in right hemisphere FA (increased white matter integrity) and a significant decrease in right hemisphere RD (decreased white matter integrity) within the thalamo-frontal tract; and a significant decrease in right hemisphere RD for the ILF. Results for the SLF did not pass the Bonferroni correction.

**Table 2:** Descriptive statistics for DTI metrics

	22q11.2DS		Control	
	*M*	*SD*	*M*	*SD*
Left hemisphere				
UF FA	.589	.036	.591	.027
UF RD	.000467	.0000424	.000471	.0000328
UF AD	.00136	.0000365	.00138	.0000342
UF number of streamlines	129.179	119.85	152.774	100.28
IFOF FA	*.691*	*.025*	*.662*	*.029*
IFOF RD	*.000373*	*.0000282*	*.000414*	*.0000352*
IFOF AD	.00147	.0000373	.00148	.0000292
IFOF number of streamlines	336.927	242.56	421.000	187.77
CB FA	.608	.032	.599	.032
CB RD	.000429	.0000338	.000448	.0000349
CB AD	.00132	.0000355	.00135	.0000350
CB number of streamlines	1228.754	398.591	1119.677	344.107
ILF FA	.645	.039	.640	.047
ILF RD	.000423	.0000535	.000430	.0000470
ILF AD	.00143	.0000514	.00146	.0000719
ILF number of streamlines	20.158	18.817	11.000	9.501
SLF FA	.643	.034	.639	.041
SLF RD	.000418	.0000498	.000420	.0000482
SLF AD	.00141	.0000496	.00141	.0000519
SLF number of streamlines	66.774	55.734	108.355	79.342
Thalamo-frontal FA	.629	.022	.624	.016
Thalamo-frontal RD	.000407	.0000218	.000447	.0000182
Thalamo-frontal AD	.00132	.0000288	.00134	.0000223
Thalamo-frontal number of streamlines	760.772	335.754	726.871	218.347
Right hemisphere				
UF FA	.601	.045	.593	.027
UF RD	.000442	.0000449	.000456	.0000295
UF AD	.00133	.0000322	.00134	.0000254
UF number of streamlines	*144.036*	*114.118*	*236.581*	*142.907*
IFOF FA	*.708*	*.027*	*.683*	*.027*
IFOF RD	*.000354*	*.0000310*	*.000386*	*.0000312*
IFOF AD	.00147	.0000359	.00148	.0000274
IFOF number of streamlines	350.255	2235.006	474.452	239.131
CB FA	*.609*	*.027*	*.588*	*.029*
CB RD	*.000421*	*.0000304*	*.000451*	*.0000310*
CB AD	.00130	.0000346	.00132	.0000298
CB number of streamlines	*995.035*	*341.383*	*818.516*	*283.127*
ILF FA	.662	.024	.646	.033
ILF RD	*.000396*	*.0000307*	*.000419*	*.0000404*
ILF AD	.00140	.0000588	.00143	.0000393
ILF number of streamlines	31.684	31.783	25.065	20.855
SLF FA	.649	.039	.656	.043
SLF RD	.000399	.0000370	.000401	.0000491
SLF AD	.00139	.0000582	.00142	.0000417
SLF number of streamlines	83.192	74.113	144.194	133.073
Thalamo-frontal FA	*.633*	*.019*	*.621*	*.023*
Thalamo-frontal RD	*.000401*	*.0000223*	*.000417*	*.0000241*
Thalamo-frontal AD	.00132	.0000226	.00132	.0000214
Thalamo-frontal number of streamlines	821.088	318.348	769.516	239.898

DTI, diffusion tensor imaging; 22q11.2DS, 22q11.2 deletion syndrome; UF, uncinate fasciculus; IFOF, inferior fronto-occipital fasciculus; CB, cingulum bundle; ILF, inferior longitudinal fasciculus; SLF, superior longitudinal fasciculus; FA, fractional anisotropy; RD, radial diffusivity; AD, axial diffusivity

Bonferroni corrected statistically significant differences indicated in italics

**Table 3:** Results of MANCOVAs for DTI Tracts

Tract	Wilks’ Lambda	*p* value	Dependent variable	*F* (df)	p value	Partial eta squared
Left UF	.899	.069	Fractional anisotropy	.004 (1, 84)	.948	.000
			Radial diffusivity	.095 (1, 84)	.758	.001
			Axial diffusivity	2.878 (1, 84)	.093	.033
			Number of streamlines	.001 (1, 84)	.976	.000
Right UF	.758	<.0001	Fractional anisotropy	1.308 (1, 84)	.256	.015
			Radial diffusivity	3.483 (1, 84)	.065	.040
			Axial diffusivity	4.344 (1, 84)	.040	.049
Left IFOF	.678	<.0001	Fractional anisotropy	26.542 (1, 83)	*<.0001*	.242
			Radial diffusivity	34.271 (1, 83)	*<.0001*	.292
			Axial diffusivity	.762 (1, 83)	.385	.009
			Number of streamlines	1.581 (1, 83)	.212	.019
Right IFOF	.681	<.0001	Axial diffusivity	1.457 (1, 83)	.231	.017
			Number of streamlines	3.111 (1, 83)	.081	.036
Left CB	.825	.003	Fractional anisotropy	4.387 (1, 85)	.039	.049
			Radial diffusivity	8.051 (1, 85)	.006	.087
			Axial diffusivity	4.091 (1, 85)	.046	.046
			Number of streamlines	8.681 (1, 85)	.004	.093
Right CB	.708	<.0001	Fractional anisotropy	14.008 (1, 85)	*<.0001*	.141
			Radial diffusivity	20.097 (1, 85)	*<.0001*	.191
			Axial diffusivity	4.739 (1, 85)	.032	.053
			Number of streamlines	19.858 (1, 85)	*<.0001*	.189
Left ILF	.810	.002	Fractional anisotropy	.181 (1, 85)	.671	.002
			Radial diffusivity	.107 (1, 85)	.744	.001
			Axial diffusivity	1.352 (1, 85)	.248	.016
			Number of streamlines	5.167 (1, 85)	.026	.057
Right ILF	.803	.001	Fractional anisotropy	7.705 (1, 85)	.007	.083
			Radial diffusivity	11.332 (1, 85)	*.001*	.118
			Axial diffusivity	5.246 (1, 85)	.024	.058
			Number of streamlines	.571 (1, 85)	.452	.007
Left SLF	.885	.046	Fractional anisotropy	.608 (1, 81)	.438	.007
			Radial diffusivity	.128 (1, 81)	.722	.002
			Axial diffusivity	.115 (1, 81)	.736	.001
			Number of streamlines	4.813 (1, 81)	.031	.056
Right SLF	.899	.072	Fractional anisotropy	.004 (1, 83)	.952	.000
			Radial diffusivity	.496 (1, 83)	.483	.006
			Axial diffusivity	2.787 (1, 83)	.099	.032
			Number of streamlines	4.252 (1, 83)	.042	.049
Left thalamo-frontal	.831	.004	Fractional anisotropy	4.347 (1, 85)	.040	.081
			Radial diffusivity	7.483 (1, 85)	.008	.059
			Axial diffusivity	.845 (1, 85)	.361	.010
			Number of streamlines	1.008 (1, 85)	.318	.012
Right thalamo-frontal	.870	.021	Fractional anisotropy	9.116 (1, 85)	*.003*	.097
			Radial diffusivity	9.911 (1, 85)	*.002*	.104
			Axial diffusivity	.731 (1, 85)	.395	.009
			Number of streamlines	2.485 (1, 85)	.119	.028

UF, uncinate fasciculus; IFOF, inferior fronto-occipital fasciculus; CB, cingulum bundle; ILF, inferior longitudinal fasciculus; SLF, superior longitudinal fasciculus

Bonferroni corrected statistically significant results indicated in italics (*p* < .004)

**Fig. 1:**
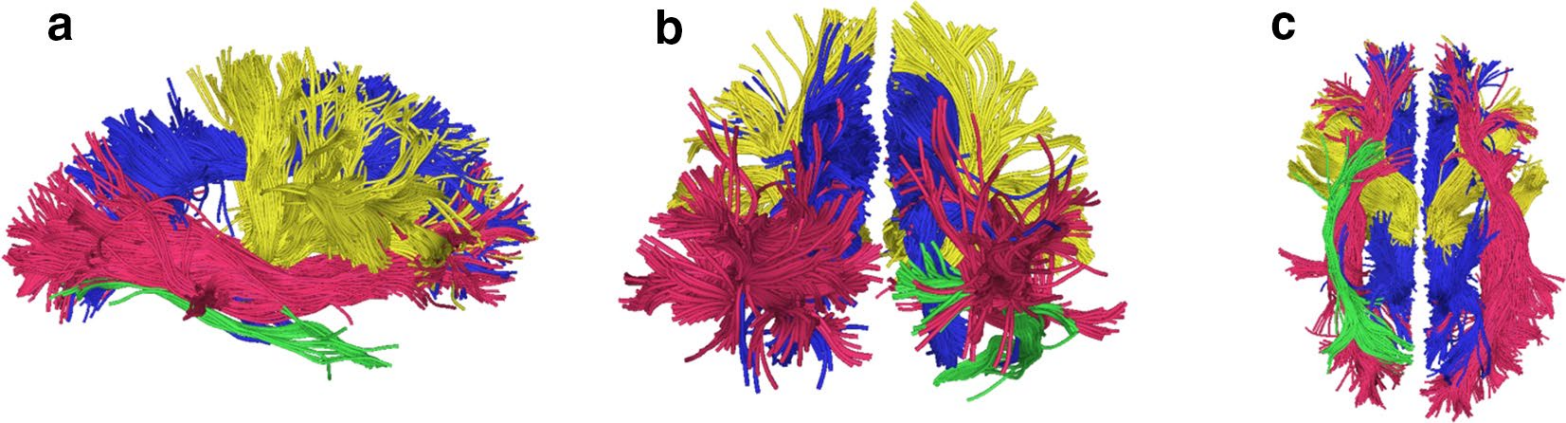
Fiber tracts of interest. ILF = *green*, IFOF = *red*, thalamo-frontal connection = *yellow*, and CB = *blue*. **a** Right lateral view (**b**) posterior view (**c**) inferior view

Nonparametric Mann–Whitney U tests were conducted for the non-normally distributed right hemisphere IFOF RD (*p* < .0001) and FA (*p* = .001), as well as the right hemisphere UF number of streamlines (*p* = .010), indicating group differences in these metrics. Compared to controls, participants with 22q11.2DS showed a significant increase in the right hemisphere IFOF FA, and a significant decrease in right hemisphere IFOF RD and UF number of streamlines. Figure 1 depicts the reconstructed white matter tracts for which we found significant differences between groups.

### Differences in social brain behavioral measures

To explore group differences in behavior-based social brain measures, we conducted an Analysis of Covariance (ANCOVA) to compare participants with 22q11.2DS and controls for scores on the SRS. We used FSIQ as a covariate to account for the fact that overall intelligence may affect social cognitive ability. Due to the nonnormality of the SRS variable, we applied a log transformation which normalized the SRS data. Descriptive statistics are presented in Table 4. Results indicated significantly higher (i.e., more impaired) scores in the group of individuals with 22q11.2DS (range = 17–140) compared to controls (range = 3–75) [*F* (1, 83) = 16.352, *p* < .0001].

**Table 4:** Descriptive statistics for social measures

Measure	22q11.2DS		Controls	
	*M*	*SD*	*M*	*SD*
SRS total	*73.321*	*3.896*	*18.433*	*5.323*
JSS social	2.930	2.412	1.700	1.841
JSS cognitive	3.982	2.066	2.900	2.057
JSS perceptual	1.298	1.773	.533	1.047
JSS impulsive	2.421	1.861	2.300	1.765
JSS physical	3.579	1.927	2.600	1.632
TEIQue emotion regulation	*3.949*	*1.196*	*5.247*	*.841*
TEIQue empathy	*3.452*	*1.043*	*5.196*	*.858*
TEIQue social awareness	*3.360*	*1.042*	*5.473*	*.965*
TEIQue emotion perception	*3.507*	*1.025*	*5.243*	*.841*

SRS, Social Responsiveness Scale, adult research version; JSS, Junior Schizotypy Scale; TEIQue, Trait Emotional Intelligence Questionnaire; 22q11.2DS, 22q11.2 deletion syndrome

Statistically significant differences indicated in italics

We conducted a multivariate analysis of variance (MANOVA) to compare scores between groups on the JSS. Descriptive statistics are presented in Table 4. Results demonstrated no significant differences between groups on the social, cognitive, physical, and perceptual subscales of the JSS; therefore, we did not run an analysis to covary for FSIQ (Table 5). We conducted a nonparametric Mann–Whitney U test for the non-normally distributed JSS Impulsive scale; results also indicated no significant differences between groups (*p* = .677).

**Table 5:** Results of MANCOVA for social measures

Measure	Wilks’ Lambda	p value	Dependent variable	*F* (df)	p value
Junior Schizotypy Scale	.934	.740	Social	1.128 (1, 31)	.296
			Cognitive	.003 (1, 31)	.958
			Perceptual	.417 (1, 31)	.523
			Physical	.016 (1, 31)	.901
TEIQue	.801	.001	Emotion regulation	5.667 (1, 83)	.020
			Empathy	14.618 (1,83)	*<.0001*
			Emotion perception	12.727 (1,83)	*.001*

TEIQue, Trait Emotional Intelligence Questionnaire

Bonferroni corrected significant results indicated in italics (*p* < .007)

Finally, we conducted a MANCOVA using FSIQ as a covariate to compare scores between groups on the TEIQue. Descriptive statistics are presented in Table 4. Due to nonnormality of the social awareness variable, we ran an ANCOVA using FSIQ as a covariate for the log transformed version of this variable. Results indicated significant differences in scores between individuals with 22q11.2DS and controls (*F* [1, 83] = 13.395, p < .0001). Therefore, parents/caregivers of individuals with 22q11.2DS rated them significantly lower than parents/caregivers of controls on three of the four facet scores of interest in this study; emotion regulation did not pass Bonferroni correction (Table 5).

### Correlations between DTI tracts and social behavioral measures

We used Pearson correlations where appropriate and Spearman correlations for variables that remained nonnormal after log transformation to examine the associations between behavioral measures of social processing and the DTI tracts that had significantly differentiated the study groups. Results are displayed in Tables 6 and 7. Among participants with 22q11.2DS, marginally significant Bonferroni-corrected associations were found between the right UF number of streamlines and the JSS Social scale (*ρ* = .260, *p* = .039), the right UF number of streamlines and the TEIQue Social Awareness facet (*ρ* = −.224, *p* = .050), and the right IFOF RD and JSS Impulsive scale (*ρ* = −.380, *p* = .006). We noted significant Bonferroni-corrected positive correlations between the right thalamo-frontal tract RD and TEIQue Empathy facet (*r* = .351, *p* = .001), as well as between the right IFOF FA and the JSS Impulsive scale (*ρ* = .412, *p* = .003) and the right UF number of streamlines and the SRS (*ρ* = .396, *p* = .001). We also found a significant Bonferroni-corrected negative correlation between the right UF number of streamlines and TEIQue Empathy facet (*ρ* = −.362, *p* = .003). There were no other significant relationships among the group of individuals with 22q11.2DS.

**Table 6:** Pearson correlation coefficients for DTI tracts and social measures

Group	SRS	JSS social	JSS cognitive	JSS perceptual	JSS physical	JSS impulsive	TEIQue emotion perception	TEIQue social awareness	TEIQue empathy	TEIQue emotion regulation
22q11.2DS										
Right CB FA	.020	.263	.114	−.193	.229	.154	−.052	.039	−.205	.032
Right CB RD	−.068	−.290	−.152	.145	−.175	−.088	.043	−.017	.265	−.070
Left CB AD	.017	−.029	−.100	−.180	.163	.157	−.177	−.033	.025	−.194
Left IFOF FA	−.079	−.067	.019	−.305	−.032	−.091	.096	.060	−.081	−.056
Left IFOF RD	.087	−.006	.025	.216	−.007	.003	−.010	.003	.071	−.062
Right ILF RD	.162	−.049	.071	.122	−.192	.126	−.141	−.127	.244	−.208
Right thalamo-frontal RD	−.130	−.084	−.041	.233	−.034	.016	.082	.059	.351*	.017
Control										
Right CB FA	−.103	.028	−.056	−.186	−.148	−.225	−.088	−.062	−.060	−.207
Right CB RD	.094	.045	.037	.236	.087	.263	.058	.069	.057	.223
Left CB AD	−.028	.335	.099	.254	.196	.209	−.070	.050	.005	.037
Left IFOF FA	.054	−.080	.061	−.003	.359	−.211	−.112	.082	−.120	−.016
Left IFOF RD	−.038	.146	−.094	.041	−.304	.212	.110	−.069	.101	.001
Right ILF RD	−.055	.112	−.060	.299	−.090	.316	.064	.125	.028	.245
Right thalamo-frontal RD	−.078	.016	−.179	.093	.105	.242	.069	.080	−.139	.016

SRS, Social Responsiveness Scale; JSS, Junior Schizotypy Scale; TEIQue, Trait Emotional Intelligence Questionnaire; CB, cingulum bundle; IFOF, inferior fronto-occipital fasciculus; ILF, inferior longitudinal fasciculus; FA, fractional anisotropy; RD, radial diffusivity; AD, axial diffusivity

* *p* < .01

**Table 7:** Spearman correlation coefficients for DTI tracts and social measures

Group	SRS	JSS Social	JSS cognitive	JSS perceptual	JSS physical	JSS impulsive	TEIQue emotion perception	TEIQue social awareness	TEIQue empathy	TEIQue emotion regulation
22q11.2DS										
Right UF number streamlines	.396**	.260*	.100	.169	.061	.133	−.172	−.224*	−.362**	−.011
Right IFOF FA	.141	.179	.153	−.163	.200	.412**	−.075	−.043	−.189	.135
Right IFOF RD	−.097	−.142	−.148	.164	−.182	−.380*	.040	.005	.201	−.169
Control										
Right UF number streamlines	−.196	.217	−.411*	−.040	.265	.071	.138	.007	.019	.180
Right IFOF FA	−.066	−.032	.084	.408	.080	.049	−.004	.120	−.046	−.085
Right IFOF RD	.107	.029	−.052	−.408	−.039	−.109	−.032	−.071	.031	.068

SRS, Social Responsiveness Scale; JSS, Junior Schizotypy Scale; TEIQue, Trait Emotional Intelligence Questionnaire; UF, uncinate fasciculus; IFOF, inferior fronto-occipital fasciculus; FA, fractional anisotropy; RD, radial diffusivity

* *p* < .05

** *p* < .01

Among the control group, we found a marginally significant negative correlation after Bonferroni correction between the right UF number of streamlines and JSS Cognitive scale (*ρ* = −.411, *p* = .017). There were no other significant correlations between DTI tracts and social brain measures among the control group.

### Associations between DTI metrics and symptoms of prodromal/overt psychosis

In order to examine whether the architecture of white matter tracts affects the development of prodromal symptoms, we ran ZIP regression analyses in order to determine whether there was a relationship between scores on a measure of prodromal symptoms (SIPS PS and SIPS NS) and DTI metrics. Results indicated that, after Bonferroni correction, several DTI metrics were significantly associated with positive symptoms of prodromal/overt psychosis, including left IFOF FA (*z* = −3.41, *p* = .001) and right ILF RD (*z* = −4.86, *p* < .0001). DTI metrics that were significantly associated with negative symptoms of prodromal/overt psychosis (after Bonferroni correction) included the right IFOF RD (*z* = −8.25; *p* < .0001) and the right IFOF FA (*z* = 7.50, *p* < .0001).

**Fig. 2:**
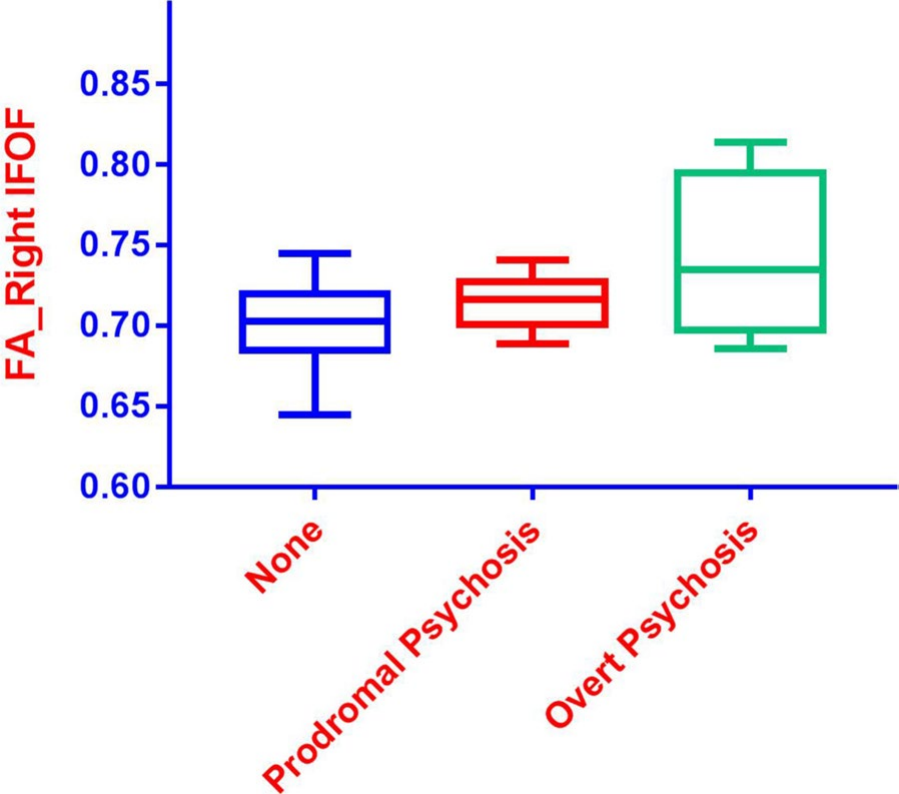
*Boxplot* of fractional anisotropy levels for the right IFOF by psychosis level. *Blue* = no psychosis, *Red* = prodromal psychosis, *Green* = overt psychosis

**Fig. 3:**
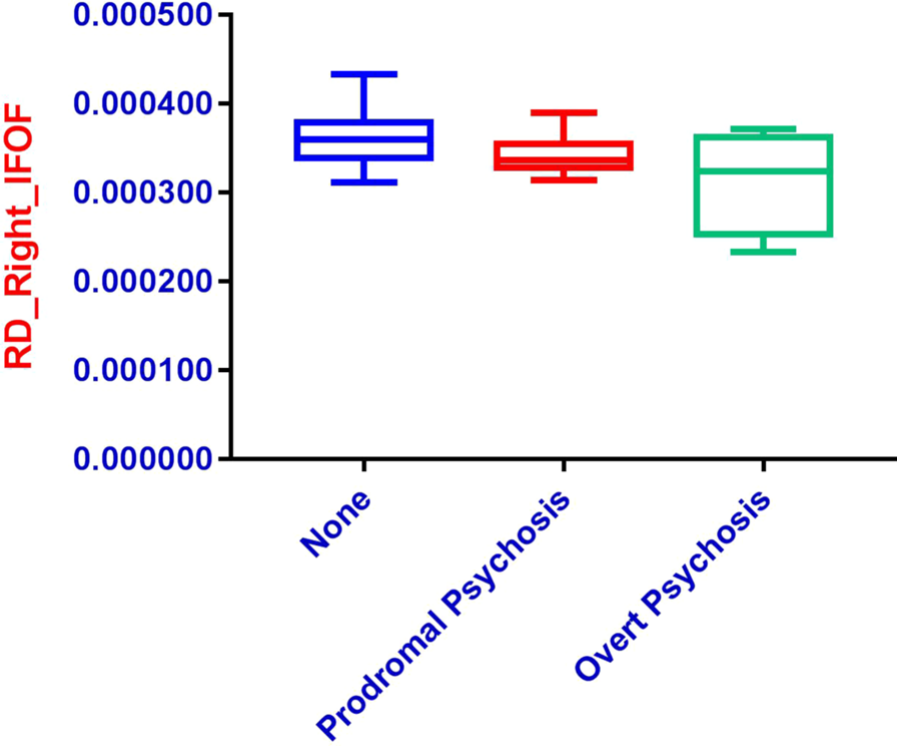
*Boxplot* of radial diffusivity levels for the right IFOF by psychosis level. *Blue* = no psychosis, *Red* = prodromal psychosis, *Green* = overt psychosis

Given these significant results, individuals with 22q11.2DS were further divided into three subgroups: those with no evidence of psychosis (22q11.2DS no psychosis); those with prodromal symptoms (22q11.2DS + prodromal psychosis) based on a score between 3 and 5 on any positive symptom item of the SIPS; and those with overt psychosis (22q11.2DS + overt psychosis) based on a diagnosis of psychotic disorder (Schizophrenia or Psychotic Disorder Not Otherwise Specified) from the SCID. Demographic information is presented in Table 8. We then conducted an exploratory MANOVA to compare individuals with 22q11.2DS with no psychosis to those with 22q11.2 and prodromal symptoms, and individuals with 22q11.2DS and overt psychosis on the DTI metrics that significantly differentiated individuals with 22q11.2DS from controls. Descriptive statistics for DTI metrics are presented in Table 9. Follow up ANOVAs indicated significant differences in the right hemisphere IFOF FA and RD (Table 10; Figs. 2, 3). Post-hoc Bonferroni-corrected analyses indicated that the group of individuals with 22q11.2DS and overt psychosis showed significant differences in DTI metrics as compared to the other two groups.

**Table 8:** Descriptive statistics for 22q11.2DS group by psychosis level

Demographic variable	22q11.2DS no psychosis (*n* = 43)	22q11.DS + prodromal psychosis (*n* = 10)	22q11.2DS + overt psychosis (*n* = 4*)*
Age [mean (SD)]	20.65 (2.15)	22.60 (2.50)	19.52 (1.76)
Gender [N (%)]			
Male	25 (58.1)	5 (50.0)	1 (25.0)
Female	18 (41.9)	5 (50.0)	3 (75.0)
FSIQ [mean (SD)]	*76.56 (12.33)*	71.00 (6.65)	*61.75 (5.32)*

22q11.2DS, 22q11.2 deletion syndrome; FSIQ, full scale IQ

Statistically significant differences indicated in italics (*p* = .015)

**Table 9:** Descriptive Statistics for DTI metrics by psychosis level

DTI metric	22q11.2DS no psychosis		22q11.DS + prodromal psychosis		22q11.2DS + overt psychosis	
	*M*	*SD*	*M*	*SD*	*M*	*SD*
RH UF number streamlines	145.488	123.879	165.000	91.459	103.750	38.638
RH IFOF FA	.702	.023	.715	.016	*.743*	*.053*
RH IFOF RD	.00036	.000026	.00034	.000023	*.00031*	*.000058*
RH CB FA	.609	.028	.062	.027	.587	.019
RH CB RD	.00042	.000031	.00041	.000030	.00043	.000032
RH ILF RD	.00039	.000028	.00038	.000039	.00039	.000035
LH IFOF FA	.692	.025	.688	.027	.679	.019
LH IFOF RD	.00037	.000028	.00037	.000032	.00038	.000026
LH CB AD	.00133	.000031	.00133	.000046	.00128	.000012

22q11.2DS, 22q11.2 deletion syndrome; RH, right hemisphere; IFOF, inferior fronto-occipital fasciculus; FA, fractional anisotropy; RD, radial diffusivity; CB, cingulum bundle; ILF, inferior longitudinal fasciculus; LH, left hemisphere; AD, axial diffusivity

Statistically significant differences identified in italics

**Table 10:** Results of MANOVA for psychosis level

Group	Wilks’ Lambda	p value	DTI Tract	*F* (df)	p value
Psychosis level	.459	.005	RH UF number streamlines	.403 (2, 52)	.670
			RH IFOF FA	5.215 (2, 52)	.009
			RH IFOF RD	5.790 (2, 52)	.005
			RH CB FA	1.882 (2,52)	.162
			RH CB RD	.587 (2, 52)	.560
			RH ILF RD	.885 (2, 52)	.419
			LH IFOF FA	.542 (2, 52)	.585
			LH IFOF RD	.040 (2, 52)	.960
			LH CB AD	3.494 (2, 52)	.038
			RH thalamo-frontal RD	1.862 (2, 52)	.166

RH, right hemisphere; UF, uncinate fasciculus; IFOF, inferior fronto-occipital fasciculus; FA, fractional anisotropy; RD, radial diffusivity; CB, cingulum bundle; ILF, inferior longitudinal fasciculus; LH, left hemisphere

## Conclusions

To our knowledge, this is the first study to use two-tensor tractography to examine the social brain among individuals with 22q11.2DS. Our results suggest significantly decreased left hemisphere RD in IFOF, and significantly increased FA in the IFOF among individuals with 22q11.2DS. We also found significantly decreased right hemisphere number of streamlines in UF, significantly increased number of streamlines in the CB, significantly decreased RD in IFOF, CB, ILF, and thalamo-frontal tract, while we found significantly increased FA in the IFOF, CB, and thalamo-frontal tract among individuals with 22q11.2DS. In addition, we found significant between group differences on social measures, particularly the SRS and TEIQue. Correlational analyses demonstrated very few associations between DTI tracts and social brain measures; the main significant findings were between the right thalamo-frontal tract RD and TEIQue Empathy facet, the right IFOF FA and the JSS Impulsive scale, the right UF number of streamlines and TEIQue Empathy facet, and the right UF number of streamlines and the SRS. Finally, ZIP regression analyses demonstrated significant associations between the presence of positive prodromal symptoms and left IFOF FA and right ILF RD. Negative symptoms were associated with the right IFOF FA and RD metrics.

### Alterations in IFOF, CB, and thalamo-frontal tract

In the present study, we found bilateral increases in FA of the IFOF, and right hemisphere increase in FA for the CB and thalamo-frontal tract among individuals with 22q11.2DS. This finding is somewhat unexpected, given previous findings of decreased FA in regions of the CB among individuals with schizophrenia [72] and individuals with 22q11.2DS [46, 73], as well as decreased FA in right hemisphere ATR/thalamo-frontal tract in individuals with ASD [53]. Interestingly, a few other studies of individuals with 22q11.2DS have demonstrated findings similar to ours. For example, Jalbrzikowski and colleagues’ [74] whole brain analyses found overall increased FA in individuals with 22q11.2DS, regardless of age. The authors attributed this finding to a combination of decreased AD and RD, noting that increases in FA have been reported in individuals with other neurodevelopmental disorders [74]. While the cause of increased FA remains unknown, possible suggestions include decreases in axonal branching [75], flattened fibers that enable increased density of white matter [76], or decreased fiber crossing [77]. In a previous set of analyses by our group based on this cohort’s assessments at the third timepoint, we found increases in bilateral FA and decreases in bilateral RD in the ALIC, as well as decreases in left hemisphere RD in the UF [45]. We suggested an overall disruption of white matter connectivity as an explanation for these findings, noting that the observed increases in FA in ALIC were driven by the lower RD levels, which in turn suggested changes in myelin development of the ALIC [45]. It is possible that a similar change affected the IFOF and CB in our current set of analyses of Time 4 data.

As stated previously, the IFOF and CB are believed to underlie visual processing/facial emotion recognition and emotional information processing, respectively, whereas the thalamo-frontal/ATR region is believed to play a role in social cognition. These are domains of known difficulty in individuals with 22q11.2DS [39, 40], as well as individuals with ASD [38, 78–80]. Intriguingly, in a study of whole brain tractography in adults with ASD, Roine and colleagues [81] found higher mean FA values for individuals with ASD compared to typically developing controls. The authors suggest the possibility that abnormal synaptic pruning may play a role in the FA increase. They note that strong physical connectivity (e.g., between synapses and tracts) and low computational connectivity (e.g., information transfer) may reinforce each other, adding to the difficulty in differentiating signal from noise [81, 82]. Alternatively, the authors suggest a more strength-based explanation. They note that that social skills and communication training prevalent in the population of individuals with ASD may lead to increased FA values in adults [81]. Research has demonstrated a link between learning a new skill and FA increases [83–85]. Many individuals with 22q11.2DS also participate in social skills or speech therapy/communication training. While these treatments were not examined in the present study, longitudinal studies that take into account the possible impact of social skills training or speech therapy interventions on white matter tracts is an area that warrants further investigation in the 22q11.2DS population.

### Impairments in behavioral measures of social processing in 22q11.2DS

Our results also demonstrated more parent-rated impairments in social processing on the SRS and TEIQue among individuals with 22q11.2DS. These results are similar to those found by other studies. For example, Jalbrzikowski and colleagues [21] found significant impairment among individuals with 22q11.2DS as compared to typically developing controls on a measure of understanding another’s intent and on an emotion recognition task [21]. Similarly, Campbell and colleagues [19] found that individuals with 22q11.2DS were less accurate than typically developing controls on measures of emotion identification and attribution [19]. Taken together, these social cognitive findings indicate that early identification of social impairments, particularly emotion identification and recognition, may provide an area for intervention in individuals with 22q11.2DS that could help to prevent or moderate future difficulty with social and adaptive functioning later in life.

### Lack of correlations between DTI metrics and measures of social processing

In contrast to Jalbrzikowski and colleagues [74], we found only a few significant correlations between social brain measures and DTI metrics (right thalamo-frontal tract RD and TEIQue Empathy, right UF number of streamlines and TEIQue Empathy, right UF number of streamlines and SRS, right IFOF FA and JSS Impulsive), whereas a few others were marginally significant. This difference may be related to the different social measures utilized between studies. For example, Jalbrzikowski and colleagues (2014) found that increased AD in the left IFOF and left UF was associated with better scores on the awareness of social inference test (TASIT) in both individuals with 22q11.2DS and controls. In addition, increased AD in these same regions was also associated with better performance on the Penn Emotion Recognition Test (ER40) in individuals with 22q11.2DS [74]. Both the TASIT and ER40 are computerized measures of social processing concepts, as opposed to the parent and self-report measures of social processing utilized in our study. Therefore, our more indirect measures may be less sensitive to social processing abilities.

Differences in our findings may also be related to age differences between the samples. Our sample consisted more of young adult participants (mean age of 22q11.2DS group = 20.87, range = 17–25), whereas participants in the Jalbrzikowski et al. study were slightly younger and had a wider age range (mean age of 22q11.2DS group = 16.3 ± 4.3). Given that the developmental trajectory of brain white matter follows a U-shaped curve, with minimum MD and RD/maximum FA levels occurring around 30 years of age [86–88], it is possible that our older sample includes a greater number of individuals who have reached those levels, and may therefore help to explain our different results.

### Associations between DTI metrics and symptoms of prodromal/overt psychosis

The presence of positive prodromal symptoms was related to DTI metrics of increased FA and in the left IFOF, and decreased RD in the right ILF, whereas presence of negative symptoms was associated with increases in FA, and decreases in RD of the IFOF. Taken together with Jalbrzikowski and colleagues [74] findings of a relationship between decreased AD in bilateral IFOF and increased positive symptom severity, these findings are intriguing, considering the IFOF’s role in visual processing/facial emotion recognition and the difficulties with these types of tasks that are seen in individuals with schizophrenia [89]. Although we cannot infer a causal relationship, these findings lend support to the possibility that disrupted axonal coherence in the IFOF may underlie social cognitive impairment and psychotic symptoms in 22q11.2DS [74]. Longitudinal DTI studies could provide further insight as to whether this white matter disruption precedes the development of prodromal symptoms in individuals with 22q11.2DS.

Within the group of individuals with 22q11.2DS, our analysis of psychosis level, while exploratory, shows evidence of a possible biomarker for psychosis in that the right hemisphere IFOF FA was significantly increased, whereas right hemisphere IFOF RD was significantly decreased in individuals with overt psychosis, as compared to those with prodromal symptoms or no psychosis.

## Limitations and suggestions

Our study does include several limitations. As previously noted, this study is a cross-sectional sample of an ongoing longitudinal study. As such, we are unable to draw any causal conclusions regarding the relationships we did find between poor social processing and positive prodromal symptoms. Longitudinal studies that follow the progress of social processing difficulties and the development of prodromal symptoms in 22q11.2DS are needed to help further elucidate this relationship. Secondly, this study did include a relatively small sample size and our groups were unequal, with fewer participants in the control group. While we did find some between group differences, the study may have suffered from reduced statistical power. As a result, differences that may have appeared with a larger sample size may not have been detected. Similarly, our comparisons between individuals with 22q11.2DS and prodromal psychosis and those with overt psychosis also likely suffered from reduced statistical power, and therefore no final conclusions can be drawn from these particular results. Our sample also included some variability, in that approximately 47% of our participants with 22q11.2DS had either prodromal symptoms of psychosis or other psychiatric diagnoses (as noted in Table 1). While not entirely certain, it is possible that this variability diluted the association between the DTI findings and behavioral measures. While these diagnoses may have affected our findings, they are common in the 22q11.2DS population, and therefore were not used as covariates in statistical analyses. Moreover, the inclusion of siblings in the control sample may have posed a limitation in that siblings of individuals with schizophrenia have been reported to show alterations in social functioning and underlying white matter connectivity, potentially affecting their control status. However, as we note in Additional file 1, sibling controls and community controls did not differ in any social behavioral or DTI measure. In addition, our measures of social processing relied on parent and self-report questionnaires, which may not be particularly sensitive to the construct of interest.

Our study is the first that we know to report on two-tensor tractography of white matter tracts in the social brain. More studies using this methodology in individuals with 22q11.2DS are needed to ensure its reliability and validity in this population. Future studies would also benefit from equally sized, larger groups. In addition, studies that combine more direct theory of mind or social cognitive measures with a two-tensor tractography approach may be more sensitive to differences in the social brain network. While quite promising with a small sample size, replication of the results within right hemisphere FA and RD and their relationship to overt psychosis is also needed. Finally, while the current study provides some important findings, longitudinal studies that track white matter development of individuals with 22q11.2DS, particularly the social brain areas of IFOF and ILF, are needed to help identify further possible biomarkers for the development of psychotic symptoms in this population.

## Additional file

**Additional file 1.** These tables depict the results of multiple analyses of variance comparing the mean scores/values between our three study groups, prior to combining the community controls and sibling controls for further analyses. The results of all post-hoc analyses are Bonferroni-corrected.

## Abbreviations

22q11.2DS: chromosome 22q11.2 deletion syndrome
VCFS: velo-cardio-facial syndrome
ADHD: attention-deficit/hyperactivity disorder
ASD: autism spectrum disorder
FA: fractional anisotropy
RD: radial diffusivity
AD: axial diffusivity
CB: cingulum bundle
ALIC: anterior limb of the internal capsule
UF: uncinate fasciculus
SLF: superior longitudinal fasciculus
IFOF: inferior fronto-occipital fasciculus
ILF: inferior longitudinal fasciculus
ATR: anterior thalamic region
fMRI: functional magnetic resonance imaging
DTI: diffusion tensor imaging
FISH: fluorescence in situ hybridization
WAIS-III: Wechsler Adult Intelligence Scale, third edition
FSIQ: full scale IQ
VIQ: verbal IQ
PIQ: performance IQ
SRS: Social Responsiveness Scale
JSS: Junior Schizotypy Scale
TEIQue: Trait Emotional Intelligence Questionnaire
SIPS: structured interview for prodromal syndromes
MPRAGE: magnetization prepared rapid acquisition gradient echo
PAT: parallel acquisition techniques
GRAPPA: generalized autocalibrating partially parallel acquisitions
FOV: field of view
DWI: diffusion weighted image
UKF: unscented Kalman filter
SPGR: spoiled gradient recalled echo
FLIRT: FMRIB’s linear image registration tool
WMQL: white matter query language
ROI: region of interest
ANOVA: analysis of variance
ANCOVA: analysis of covariance
MANOVA: multivariate analysis of variance
MANCOVA: multivariate analysis of covariance
ZIP: zero-inflated Poisson
TASIT: the awareness of social inference test
ER40: Penn Emotional Recognition Test
