# Supplementary material for: The social brain network in 22q11.2 deletion syndrome: a diffusion tensor imaging study
Source: Behav Brain Funct. 2017 Feb 16;13:4. doi: 10.1186/s12993-017-0122-7 (PMC5314621; doi:10.1186/s12993-017-0122-7)
Supplement: Supplementary file 1 — Additional file 1. These tables depict the results of multiple analyses of variance comparing the mean scores/values between our three study groups, prior to combining the community controls and sibling controls for further analyses. The results of all post-hoc analyses are Bonferroni-corrected. [file 12993_2017_122_MOESM1_ESM.docx]

**Supplementary Information**

Results of Bonferroni Post-hoc Procedures for Social Measures

| Dependent Variable | (I) Group | (J) Group | Mean Difference  (I-J) | Std. Error | *p* |
| --- | --- | --- | --- | --- | --- |
| SRS Total Score | Community Control | Sibling Control | -1.639 | 10.929 | 1.000 |
|  |  | 22q11.2DS | -55.544* | 7.946 | < .0001 |
|  | Sibling Control | 22q11.2DS | -53.905* | 9.329 | < .0001 |
| JSS Social | Community Control | Sibling Control | -.361 | .836 | 1.000 |
|  |  | 22q11.2DS | -1.374 | .607 | .078 |
|  | Sibling Control | 22q11.2DS | -1.013 | .713 | .477 |
| JSS Cognitive | Community Control | Sibling Control | .389 | .772 | 1.00 |
|  |  | 22q11.2DS | -.927 | .560 | .305 |
|  | Sibling Control | 22q11.2DS | -1.316 | .658 | .146 |
| JSS Perceptual | Community Control | Sibling Control | .056 | .588 | 1.000 |
|  |  | 22q11.2DS | -.743 | .427 | .257 |
|  | Sibling Control | 22q11.2DS | -.798 | .501 | .345 |
| JSS Impulsive | Community Control | Sibling Control | -.472 | .684 | 1.000 |
|  |  | 22q11.2DS | -.310 | .496 | 1.000 |
|  | Sibling Control | 22q11.2DS | .162 | .583 | 1.000 |
| JSS Physical | Community Control | Sibling Control | .861 | .680 | .627 |
|  |  | 22q11.2DS | -.635 | .493 | .606 |
|  | Sibling Control | 22q11.2DS | -1.496* | .580 | .035 |
| TEIQue Emotion Regulation | Community Control | Sibling Control | .528 | .403 | .582 |
|  |  | 22q11.2DS | 1.509* | .293 | < .0001 |
|  | Sibling Control | 22q11.2DS | .981* | .344 | .016 |
| TEIQue Emotion Perception | Community Control | Sibling Control | .614 | .355 | .264 |
|  |  | 22q11.2DS | 1.982* | .258 | < .0001 |
|  | Sibling Control | 22q11.2DS | 1.368* | .303 | < .0001 |
| TEIQue Empathy | Community Control | Sibling Control | .173 | .368 | 1.000 |
|  |  | 22q11.2DS | 1.813* | .268 | < .0001 |
|  | Sibling Control | 22q11.2DS | 1.640* | .314 | < .0001 |
| TEIQue Social Awareness | Community Control | Sibling Control | .068 | .381 | 1.000 |
|  |  | 22q11.2DS | 2.140* | .277 | < .0001 |
|  | Sibling Control | 22q11.2DS | 2.071* | .325 | < .0001 |

*Note.* 22q11.2DS = 22q11.2 Deletion Syndrome; SRS = Social Responsiveness Scale; JSS- = Junior Schizotypy Scale; TEIQue = Trait Emotional Intelligence Questionnaire.

Results of Bonferroni Post-hoc Procedures for DTI Metrics – Cingulum Bundle

| Dependent Variable | (I) Group | (J) Group | Mean Difference  (I-J) | Std. Error | *p* |
| --- | --- | --- | --- | --- | --- |
| Left CB FA | Community Control | Sibling Control | .011 | .012 | 1.000 |
|  |  | 22q11.2DS | -.005 | .009 | 1.000 |
|  | Sibling Control | 22q11.2DS | -.016 | .010 | .369 |
| Left CB RD | Community Control | Sibling Control | -.000013 | .000013 | .886 |
|  |  | 22q11.2DS | .000013 | .000009 | .427 |
|  | Sibling Control | 22q11.2DS | .000027* | .000011 | .048 |
| Left CB AD | Community Control | Sibling Control | .00000023 | .000013 | 1.000 |
|  |  | 22q11.2DS | .000024* | .0000094 | .033 |
|  | Sibling Control | 22q11.2DS | -.000025 | .000011 | .095 |
| Left CB Number of Streamlines | Community Control | Sibling Control | -194.548 | 139.529 | .501 |
|  |  | 22q11.2DS | -184.386 | 100.241 | .208 |
|  | Sibling Control | 22q11.2DS | 10.162 | 120.185 | 1.000 |
| Right CB FA | Community Control | Sibling Control | -.00436 | .010 | 1.000 |
|  |  | 22q11.2DS | -.022* | .007 | .012 |
|  | Sibling Control | 22q11.2DS | -.018 | .009 | .155 |
| Right CB RD | Community Control | Sibling Control | .00000017 | .000011 | 1.000 |
|  |  | 22q11.2DS | .000029* | .0000082 | .001 |
|  | Sibling Control | 22q11.2DS | .000028* | .0000097 | .016 |
| Right CB AD | Community Control | Sibling Control | -.000013 | .000012 | .886 |
|  |  | 22q11.2DS | .000016 | .0000087 | .228 |
|  | Sibling Control | 22q11.2DS | .000028* | .000010 | .024 |
| Right CB Number of Streamlines | Community Control | Sibling Control | -76.649 | 119.213 | 1.000 |
|  |  | 22q11.2DS | -207.351 | 85.645 | .053 |
|  | Sibling Control | 22q11.2DS | -127.702 | 102.685 | .651 |

*Note.* 22q11.2DS = 22q11.2 Deletion Syndrome; CB = Cingulum Bundle; FA = Fractional Anisotropy; RD = Radial Diffusivity; AD = Axial Diffusivity.

Results of Bonferroni Post-hoc Procedures for DTI Metrics - Inferior Longitudinal Fasciculus

| Dependent Variable | (I) Group | (J) Group | Mean Difference  (I-J) | Std. Error | *p* |
| --- | --- | --- | --- | --- | --- |
| Left ILF FA | Community Control | Sibling Control | .012 | .016 | 1.000 |
|  |  | 22q11.2DS | -.00001 | .011 | 1.000 |
|  | Sibling Control | 22q11.2DS | -.012 | .013 | 1.000 |
| Left ILF RD | Community Control | Sibling Control | -.000021 | .000019 | .803 |
|  |  | 22q11.2DS | -.0000091 | .0000014 | 1.000 |
|  | Sibling Control | 22q11.2DS | .000020 | .000016 | .656 |
| Left ILF AD | Community Control | Sibling Control | -.000011 | .000022 | 1.000 |
|  |  | 22q11.2DS | .000019 | .000016 | .628 |
|  | Sibling Control | 22q11.2DS | .000031 | .000019 | .322 |
| Left ILF Number of Streamlines | Community Control | Sibling Control | -1.768 | 6.001 | 1.000 |
|  |  | 22q11.2DS | -9.842 | 4.311 | .075 |
|  | Sibling Control | 22q11.2DS | -8.075 | 5.167 | .366 |
| Right ILF FA | Community Control | Sibling Control | .015 | .010 | .440 |
|  |  | 22q11.2DS | -.010 | .007 | .517 |
|  | Sibling Control | 22q11.2DS | -.025* | .009 | .017 |
| Right ILF RD | Community Control | Sibling Control | -.000016 | .000013 | .662 |
|  |  | 22q11.2DS | .000017 | .0000091 | .186 |
|  | Sibling Control | 22q11.2DS | .000033* | .0000011 | .010 |
| Right ILF AD | Community Control | Sibling Control | .000012 | .000019 | 1.000 |
|  |  | 22q11.2DS | .000016 | .0000087 | .109 |
|  | Sibling Control | 22q11.2DS | .000018 | .000017 | .886 |
| Right ILF Number of Streamlines | Community Control | Sibling Control | -6.013 | 10.532 | 1.000 |
|  |  | 22q11.2DS | -8.947 | 7.567 | .721 |
|  | Sibling Control | 22q11.2DS | -2.934 | 9.072 | 1.00 |

*Note.* 22q11.2DS = 22q11.2 Deletion Syndrome; ILF = Inferior Longitudinal Fasciculus; FA = Fractional Anisotropy; RD = Radial Diffusivity; AD = Axial Diffusivity.

Results of Bonferroni Post-hoc Procedures for DTI Metrics - Superior Longitudinal Fasciculus

| Dependent Variable | (I) Group | (J) Group | Mean Difference  (I-J) | Std. Error | *p* |
| --- | --- | --- | --- | --- | --- |
| Left SLF FA | Community Control | Sibling Control | .002 | .014 | 1.000 |
|  |  | 22q11.2DS | -.004 | .009 | 1.000 |
|  | Sibling Control | 22q11.2DS | -.005 | .012 | 1.000 |
| Left SLF RD | Community Control | Sibling Control | -.0000071 | .000018 | 1.000 |
|  |  | 22q11.2DS | -.0000006 | .0000013 | 1.000 |
|  | Sibling Control | 22q11.2DS | .0000065 | .000016 | 1.000 |
| Left SLF AD | Community Control | Sibling Control | -.000011 | .000019 | 1.000 |
|  |  | 22q11.2DS | -.0000042 | .000014 | 1.000 |
|  | Sibling Control | 22q11.2DS | .0000073 | .000016 | 1.000 |
| Left SLF Number of Streamlines | Community Control | Sibling Control | -27.702 | 24.055 | .759 |
|  |  | 22q11.2DS | 30.858 | 17.444 | .242 |
|  | Sibling Control | 22q11.2DS | 58.560* | 20.856 | .019 |
| Right SLF FA | Community Control | Sibling Control | .008 | .015 | 1.00 |
|  |  | 22q11.2DS | .010 | .011 | 1.00 |
|  | Sibling Control | 22q11.2DS | .002 | .013 | 1.000 |
| Right SLF RD | Community Control | Sibling Control | -.000014 | .000015 | 1.000 |
|  |  | 22q11.2DS | -.0000039 | .0000011 | 1.000 |
|  | Sibling Control | 22q11.2DS | .0000097 | .0000013 | 1.000 |
| Right SLF AD | Community Control | Sibling Control | -.0000078 | .000019 | 1.000 |
|  |  | 22q11.2DS | .000025 | .0000014 | .250 |
|  | Sibling Control | 22q11.2DS | .000033 | .000017 | .172 |
| Right SLF Number of Streamlines | Community Control | Sibling Control | 39.746 | 36.567 | .841 |
|  |  | 22q11.2DS | 76.397* | 26.389 | .015 |
|  | Sibling Control | 22q11.2DS | 36.652 | 31.596 | .748 |

*Note.* 22q11.2DS = 22q11.2 Deletion Syndrome; SLF = Superior Longitudinal Fasciculus; FA = Fractional Anisotropy; RD = Radial Diffusivity; AD = Axial Diffusivity.

Results of Bonferroni Post-hoc Procedures for DTI Metrics - Uncinate Fasciculus

| Dependent Variable | (I) Group | (J) Group | Mean Difference  (I-J) | Std. Error | *p* |
| --- | --- | --- | --- | --- | --- |
| Left UF FA | Community Control | Sibling Control | -.001 | .012 | 1.000 |
|  |  | 22q11.2DS | .002 | .009 | 1.000 |
|  | Sibling Control | 22q11.2DS | .003 | .011 | 1.000 |
| Left UF RD | Community Control | Sibling Control | -.00000042 | .000015 | 1.000 |
|  |  | 22q11.2DS | .0000035 | .000010 | 1.000 |
|  | Sibling Control | 22q11.2DS | .0000039 | .000013 | 1.000 |
| Left UF AD | Community Control | Sibling Control | -.0000074 | .000013 | 1.000 |
|  |  | 22q11.2DS | .000017 | .0000095 | .247 |
|  | Sibling Control | 22q11.2DS | .000024 | .000011 | .111 |
| Left UF Number of Streamlines | Community Control | Sibling Control | -45.373 | 41.745 | .841 |
|  |  | 22q11.2DS | 6.032 | 30.057 | 1.000 |
|  | Sibling Control | 22q11.2DS | 51.405 | 36.013 | .472 |
| Right UF FA | Community Control | Sibling Control | -.008 | .015 | 1.00 |
|  |  | 22q11.2DS | -.011 | .010 | .897 |
|  | Sibling Control | 22q11.2DS | .003 | .013 | 1.000 |
| Right UF RD | Community Control | Sibling Control | .0000084 | .000015 | 1.000 |
|  |  | 22q11.2DS | .000018 | .0000011 | .308 |
|  | Sibling Control | 22q11.2DS | .0000093 | .0000013 | 1.000 |
| Right UF AD | Community Control | Sibling Control | .00000018 | .000011 | 1.000 |
|  |  | 22q11.2DS | .000016 | .0000080 | .167 |
|  | Sibling Control | 22q11.2DS | .000015 | .0000096 | .339 |
| Right UF Number of Streamlines | Community Control | Sibling Control | -13.737 | 46.355 | 1.000 |
|  |  | 22q11.2DS | 87.227* | 33.377 | .032 |
|  | Sibling Control | 22q11.2DS | 100.964* | 39.990 | .040 |

*Note.* 22q11.2DS = 22q11.2 Deletion Syndrome; UF = Uncinate Fasciculus; FA = Fractional Anisotropy; RD = Radial Diffusivity; AD = Axial Diffusivity.

Results of Bonferroni Post-hoc Procedures for DTI Metrics– Inferior Fronto-Occipital Fasciculus

| Dependent Variable | (I) Group | (J) Group | Mean Difference  (I-J) | Std. Error | *p* |
| --- | --- | --- | --- | --- | --- |
| Left IFOF FA | Community Control | Sibling Control | .006 | .010 | 1.000 |
|  |  | 22q11.2DS | -.027* | .007 | .001 |
|  | Sibling Control | 22q11.2DS | -.033* | .009 | .001 |
| Left IFOF RD | Community Control | Sibling Control | -.0000096 | .000011 | 1.000 |
|  |  | 22q11.2DS | .000037* | .0000082 | < .0001 |
|  | Sibling Control | 22q11.2DS | .000047* | .0000099 | < .0001 |
| Left IFOF AD | Community Control | Sibling Control | -.0000064 | .000013 | 1.000 |
|  |  | 22q11.2DS | .000012 | .0000092 | .585 |
|  | Sibling Control | 22q11.2DS | .000018 | .000011 | .297 |
| Left IFOF Number of Streamlines | Community Control | Sibling Control | -44.868 | 83.144 | 1.000 |
|  |  | 22q11.2DS | 66.704 | 60.003 | .808 |
|  | Sibling Control | 22q11.2DS | 111.573 | 71.843 | .373 |
| Right IFOF FA | Community Control | Sibling Control | .014 | .010 | .497 |
|  |  | 22q11.2DS | -.020* | .007 | .021 |
|  | Sibling Control | 22q11.2DS | -.034* | .009 | .001 |
| Right IFOF RD | Community Control | Sibling Control | -.000015 | .000011 | .579 |
|  |  | 22q11.2DS | .000027* | .0000082 | .006 |
|  | Sibling Control | 22q11.2DS | .000041* | .0000099 | < .0001 |
| Right IFOF AD | Community Control | Sibling Control | .0000095 | .000012 | 1.000 |
|  |  | 22q11.2DS | .000012 | .0000088 | .519 |
|  | Sibling Control | 22q11.2DS | .0000026 | .000011 | 1.000 |
| Right IFOF Number of Streamlines | Community Control | Sibling Control | -62.215 | 87.459 | 1.000 |
|  |  | 22q11.2DS | 100.114 | 63.117 | .350 |
|  | Sibling Control | 22q11.2DS | 162.329 | 75.571 | .104 |

*Note.* 22q11.2DS = 22q11.2 Deletion Syndrome; IFOF = Inferior Fronto-Occipital Fasciculus; FA = Fractional Anisotropy; RD = Radial Diffusivity; AD = Axial Diffusivity.

Results of Bonferroni Post-hoc Procedures for DTI Metrics – Thalamo-frontal Tract

| Dependent Variable | (I) Group | (J) Group | Mean Difference  (I-J) | Std. Error | *p* |
| --- | --- | --- | --- | --- | --- |
| Left thalamo-frontal FA | Community Control | Sibling Control | .008 | .007 | .754 |
|  |  | 22q11.2DS | -.002 | .005 | 1.000 |
|  | Sibling Control | 22q11.2DS | -.011 | .006 | .285 |
| Left thalamo-frontal RD | Community Control | Sibling Control | -.0000097 | .0000076 | .616 |
|  |  | 22q11.2DS | .0000069 | .0000054 | .625 |
|  | Sibling Control | 22q11.2DS | .000017* | .0000065 | .039 |
| Left thalamo-frontal AD | Community Control | Sibling Control | -.0000039 | .0000099 | 1.000 |
|  |  | 22q11.2DS | .0000098 | .0000071 | .513 |
|  | Sibling Control | 22q11.2DS | .000014 | .0000085 | .335 |
| Left thalamo-frontal Number of Streamlines | Community Control | Sibling Control | -29.307 | 111.246 | 1.000 |
|  |  | 22q11.2DS | -45.246 | 79.922 | 1.000 |
|  | Sibling Control | 22q11.2DS | -15.939 | 95.823 | 1.000 |
| Right thalamo-frontal FA | Community Control | Sibling Control | .010 | .008 | .577 |
|  |  | 22q11.2DS | -.008 | .006 | .446 |
|  | Sibling Control | 22q11.2DS | -.018* | .007 | .022 |
| Right thalamo-frontal RD | Community Control | Sibling Control | -.000010 | .0000084 | .675 |
|  |  | 22q11.2DS | .000011 | .0000061 | .208 |
|  | Sibling Control | 22q11.2DS | .000021* | .0000073 | .012 |
| Right thalamo-frontal AD | Community Control | Sibling Control | .00000090 | .0000082 | 1.000 |
|  |  | 22q11.2DS | .0000072 | .0000059 | .676 |
|  | Sibling Control | 22q11.2DS | .0000081 | .0000071 | .765 |
| Right thalamo-frontal Number of Streamlines | Community Control | Sibling Control | 58.627 | 108.626 | 1.000 |
|  |  | 22q11.2DS | -28.877 | 78.039 | 1.000 |
|  | Sibling Control | 22q11.2DS | -87.504 | 93.565 | 1.000 |

*Note.* 22q11.2DS = 22q11.2 Deletion Syndrome; FA = Fractional Anisotropy; RD = Radial Diffusivity; AD = Axial Diffusivity.
